# Assessment of northern bobwhite survival and fitness in the West Gulf Coastal Plain ecoregion

**DOI:** 10.1371/journal.pone.0200544

**Published:** 2018-07-17

**Authors:** Jacob W. Doggett, Alexandra Locher

**Affiliations:** 1 Montana Fish, Wildlife & Parks, Region 4 Headquarters, Great Falls, Montana, United States of America; 2 Biology Department, Grand Valley State University, Allendale, Michigan, United States of America; Sichuan University, CHINA

## Abstract

In the West Gulf Coastal Plains (WGCP) northern bobwhite (*Colinus virginianus)* are declining faster than range-wide averages and such declines have been linked to the consequences of land management. Management for the endangered red-cockaded woodpecker (*Picoides borealis*) has benefitted northern bobwhite by restoring mature pine-grassland ecosystems in some areas of the region. However, at Felsenthal National Wildlife Refuge, Crossett, Arkansas, USA, the bobwhite population was not increasing despite the availability of seemingly suitable habitat from management for the endangered species. To understand factors that may be affecting bobwhite survival on Felsenthal National Wildlife Refuge we conducted a telemetry study and assessed summer survival, brood survival, and nest success from 1 April– 11 August in 2013 and 1 April– 15 August in 2014. We also calculated home-range sizes and measured microhabitat characteristics around nests. Summer survival rates were 71% (SE = 0.17) and 47% (SE = 0.14); while nest success was 47% (SE = 0.02) and 100% for 2013 and 2014, respectively. Between years, both 95% and 50% kernel home-ranges were not different (pooled, 63.92±6.07 ha and 14.94±1.75 ha); however minimum convex polygon home-range sizes were (113.8 ± 20.1 ha in 2013; and 393.1 ± 49.0 ha in 2014, P < 0.001). Only numerical differences in microhabitat vegetation characteristics of nest sites and non- nest sites were observed. We suggest management for red-cockaded woodpeckers supports bobwhite populations but only as a buffer against more severe declines. Since bobwhites are declining range-wide, we believe areas federally managed for red-cockaded woodpeckers will become increasingly more important for sustaining regional bobwhite population levels.

## Introduction

In the West Gulf Coastal Plains (WGCP), an ecoregion covering parts of Louisiana, Arkansas, Texas and Oklahoma, bobwhites are experiencing declines steeper than range-wide averages [1,2]. In 2015, the Breeding Bird Survey (BBS) showed regional indices had declined 5.5% annually since the 1960s [3]. Restoration in the WGCP is constrained by industrial/corporate ownership of forestlands, past introduction of sod-forming grasses, and private land ownership patterns that are fragmented into small parcels [4]. In their 2011 report, the Northern Bobwhite Technical Committee [4] suggested the best opportunities for restoring bobwhite populations in the WGCP include pine and oak savanna restoration, increased use of prescribed fire, restoration of warm season grasses, and improved management of existing conservation lands.

Many state and federally–owned conservation lands that occur throughout the WGCP are managed for red-cockaded woodpeckers (*Picoides borealis*; hereafter RCW) [5]; a federally endangered species endemic to the mature pine ecosystems of the southeastern United States [6,7]. Lands under RCW management are important because management for RCWs is designed to restore mature pine-grassland ecosystems [5] and has been reported to benefit early successional species like RCW and northern bobwhite [8–11]. Previous research suggested that connecting isolated patches of suitable habitat through RCW management practices could lead to increased bobwhite abundance and regional population stability [12]; thus where RCW management is occurring, bobwhite populations could potentially be conserved. Several conservation areas across the WGCP, support populations of bobwhites [13–15]; however, not all of these populations are growing [15].

In southeast Arkansas, Felsenthal National Wildlife Refuge, hereafter Felsenthal NWR, reported declining bobwhite populations despite seemingly ideal habitat conditions. Mention of decline on Felsenthal NWR was surprising because bobwhites are considered a species of concern under the management actions appropriated for RCW [16] and such management is known to benefit them. If RCW management indeed benefits bobwhites, management for RCW may not only provide refuge for declining populations; but also, restrictions resulting from the legal ramifications of land stewardship responsibilities for RCW could become more easily accepted when the popular upland game bird species is also supported [17]. Evidence suggesting bobwhites are not responding to RCW management on Felsenthal NWR warranted an investigation.

For bobwhites, site specific, descriptive data on survival and mortality are generally prerequisite for the development of sound management strategies [18]. In declining populations of bobwhites, survival comprises the greatest contribution to variation in rates of population change [19,20]. Specifically, summer survival, nest success and chick survival are important metrics to understand bobwhite population dynamics [19,21]. In addition to fecundity and nest success, bobwhites require a unique subset of habitat characteristics to thrive. The most important characteristics are those required for nesting and brood rearing [1,22–24]. The overarching goals of the project were to understand the current status of the bobwhite population at Felsenthal NWR in response to management practices for RCW, and understand specific vegetation structure and composition contributing to nest success. Our specific objectives were to 1) quantify northern bobwhite survival rates during the nesting and brood-rearing periods; 2) quantify vegetation conditions associated with bobwhite nest success; and 3) identify other potential factors contributing to declines in southern Arkansas. Understanding bobwhite population dynamics at Felsenthal NWR is not only useful for managing bobwhites and RCW together, it also may enhance understanding of the current population dynamics in the West Gulf Coastal Plains–an area where research is lacking and bobwhite declines have been reported as severe.

## Materials and methods

### Study area

Felsenthal NWR lies across portions of Ashley, Bradley and Union Counties (33°7'52.4437"N, 92°11'26.3253"W) in southeastern Arkansas. The refuge comprises approximately 16,000 ha of bottomland hardwood forests, 4,000 ha of upland forest and a fluctuating 6,000 to 14,000-ha lock and dam-controlled reservoir. Land use surrounding the refuge is heavily managed for timber production including pulpwood, poles and saw logs [16]; soil types in the upland areas range from Una silty clay loam to Guyton loam [25]. The area we chose for the assessment represents the largest spatially distinct upland area on Felsenthal NWR and comprised approximately 60% of the upland area on the refuge and 10% of Felsenthal NWR’s total area (approximately 3,100 ha). The study area is dominated by loblolly pine (*Pinus taeda*) intermixed with white oak (*Quercus alba)*, post oak (*Quercus stellata*), southern red oak (*Quercus falcata*), cherry bark oak (*Quercus pagoda)*, common persimmon (*Diospyros virginiana*), and sweet gum (*Liquidambar styraciflua*). Hardwood canopy cover in areas managed specifically for RCW represented < 30% overall composition. Management consisted of prescribed burns every 3–5 years, even-aged timber management (100-year rotation), and single tree harvests to attain a basal area between 13.7–16.1 m^2^/ha [26]. Understory plant communities include a variety of woody and herbaceous species. Woody species included dewberry (*Rubus* spp.), deerberry (*Vaccinium angustifolium*), greenbrier (*Smilax* spp.), American beauty berry (*Callicarpa americana*), and smooth sumac (*Rhus glabra*). Common graminoids are slender wood oats (*Chasmanthium laxum*), indian wood oats (*Chasmanthium latifolium*), broomsedge bluestem (*Andropogon virginicus*), switchgrass (*Panicum virgatum*), and various sedges (Family Cyperaceae).

We chose the area because of its potential for holding sufficient bobwhite numbers to conduct the study. Weil (2012) created a habitat suitability model that described the area as having distinct spatial patterns of low to medium–density pine as well as grass components that predicted the highest chances for bobwhite presence on the refuge [26]. In addition, reports and observations by both Weil (2012) and refuge staff suggested the area supported several coveys which we would be able to monitor (Rick Eastridge, U.S. Fish and Wildlife Service, personal communication). Based on the management practices for RCW, these upland areas were thought to be suitable for bobwhite as well.

### Trapping

To find nests and assess survival on Felsenthal NWR, we trapped wild bobwhites continuously from March through August in 2013 and from March through May in 2014 using baited funnel traps (checked ≥ 2 times daily) and mist nets. Mist nets were deployed in two different fashions to either, call in individuals using audio and decoy lures, or to opportunistically capture individuals whose location was already known [27]. Captured birds were banded with two aluminum leg bands and fitted with 6.5-g pendulum style radio-transmitters equipped with 14-hr mortality censors (American Wildlife Enterprises, Monticello, FL). During primary capture events, we recorded each individual’s sex, age, weight and condition and fitted them with transmitters if they were in visually good condition and weighed ≥ 130 g [28]. All birds captured together were released together from their capture location. All of our capture, handling and release methods were approved by the Institutional Animal Care and Use Committee through Grand Valley State University (Project # 12-06-A), Arkansas Game and Fish Commission (AGFC Code 15–30), and the U.S. Fish and Wildlife Service (Permit #43579-2013-017).

To improve trapping success in 2014, we released 60 radio-marked, pen-reared northern bobwhite in addition to normal trapping efforts in the month of March. Research suggested that during large releases of pen-reared bobwhites, resident wild bobwhites would occasionally be attracted to the area [29,30]. Pen-reared birds were purchased and transported from Ozark Quail Farms (Republic, MO) and consisted of an equal sex ratio of 13–16-wk old flight-capable individuals. Prior to release, all pen-reared quail were kept in an approximately 9.29-m^2^ holding pen covered in 2.54-cm nylon mesh and fed a mixture of game starter, cracked corn, milo and wheat. Because of the coordination involved with processing (i.e., banding, weighing, and transmitter fixing) as well as releasing pen-reared bobwhites, we randomly selected individuals that were acclimated to transmitters for a range of 1–20 days before being released. We released all of the pen-reared bobwhites in groups of 6–9 individuals (9 groups) and varied group composition by sex ratio, age ratio, and transmitter acclimation. We chose release sites we arbitrarily selected as having relatively higher quality habitat conditions than areas not chosen for release. All of these sites were scattered with 0.45–2.27 kg of feed prior to release and monitored for approximately 30-min post release. Once radio-marked pen-reared quail were released, we tracked them via telemetry daily and made efforts to visually observe the group from approximately 10-m during each visit.

### Tracking

In both 2013 and 2014, we tracked all marked wild birds ≥ 5 times per week via the homing method [31]. The homing method consists of approaching a marked bird to a distance between 10–50 m and then circling the individual to accurately estimate its location. We estimated each individual’s daily location using a 2-step protocol whereby we recorded the Universal Transverse Mercator (UTM) coordinates from a Garmin GPSMap 62sc Global Positioning System (GPS) receiver (Garmin GPS, Garmin International, Inc., Olathe, KS) at the observers’ position, and then measured the azimuth and estimated distance to the radio-marked individuals. We also recorded date and time. During each observation, we made every effort to minimize flushing radio-marked individuals except when it was useful to identify unmarked individuals with radio-marked bobwhite. When mortality signals were triggered we approached the location of the transmitter to investigate cause of death. In instances where marked birds were found dead, we assessed the cause of mortality based on transmitter damage, remains, and physical evidence at the site [32,33].

When bobwhites were tracked to the same location ≥ 2 consecutive days during the nesting season we assumed that there was a nest present [34,35]. We noted potential nest locations and visited them immediately the next day to confirm the location and presence of the marked bird. Potential nest sites were investigated only when the bird was determined to be temporarily away [36]; and since bobwhites typically do not spend much time at the nest until the onset of incubation, we assumed the parent’s daily presence on the nest marked the end of the egg laying period and beginning of incubation [21,22,37]. When visiting nests for the first time, we installed either one or two motion-activated cameras at the site (Primos Ultra-blackout Truth Cam). In the event of a failed nest, these cameras allowed identification of the cause, and date and time of failure. We placed all cameras between 1-m and 5-m away from the nest and camouflaged them to avoid attracting predators [38].

To accurately record nest success, we tracked incubating adults to the nest daily. During this time, we made visual observations of the nest only when the nest was suspected to have been predated, or to exchange batteries and SD cards. Routine camera maintenance was necessary about every two to four days, but was only completed when the incubating adult was temporarily away. During visits to the nest sites, we wore rubber boots and gloves to avoid leaving scent that might attract potential predators. We documented nests as active, successful or failed. We visited hatched nests only when telemetry indicated the adult and brood were away from the nest >50 m and documented nests as successful if the incubating adult remained at the nest throughout the incubation period and hatched ≥1 egg. During occasions when the incubating adult did not remain at the nest and/or in which ≥1 egg was predated, we documented these nests as abandoned or failed, respectively. When nests were predated, we recorded the predator species responsible for the nest predation based on camera photos as well as the diagnostic guidelines [38,39].

To monitor brood survival, we tracked brooding parents daily and to within 50 meters. Since bobwhite chicks are unable to fly until 14 days of age [40], we did not flush chicks until 14 days after they hatched. In addition, we conducted follow-up flushes at 21, 28, 35 and 42 days post-hatch, respectively [41]. Because bobwhite chicks typically become independent between 21 and 42 days post hatch and brooding parents are known to abandon chicks during this time as well [22], these procedures allowed us to record the number surviving until brooding was complete.

### Vegetation sampling

To quantify microhabitat, we measured characteristics at nest sites within one week after the nest had been vacated to avoid creating negative consequences for the brood. We used 0.04-ha circular plots at the nest location and paired it with an equally sized non-nest plot within a randomly chosen distance between 0 m and 200 m away and in a random direction. Sampling vegetation at random points allowed for vegetation at the nest site to be compared with available vegetation conditions throughout the study area [35,42]. For each plot, we described vegetation characteristics by percent ground coverage, horizontal ground cover density (i.e., vertical structure), tree basal area, stem density, tallest vegetation height over the nest and percent overstory tree canopy cover.

To estimate percent ground coverage at each location we took 13 visual estimates from a 1-m^2^ quadrat. Measurements were taken over the nest and also 1.5, 3.5 and 5.5 m from the nest in each of the four cardinal directions. We based these measurements off of Daubenmire’s (1959) midpoint values which consisted of categorizing cover types into five coverage classes to estimate the categorical frequency and composition of available vegetation [43]. For each of these measurements, we chose the categories: graminoids, forbs, bare ground, litter and woody vegetation because they are critical components for bobwhite nesting habitat [40,44–46]. Each percent ground coverage measurement was visually estimated from height of approximately 1.37 m above the ground.

To assess nest cover suitability, we measured horizontal ground cover density using a vertical profile cloth sheet with a 10-cm grid, 1-m wide by 2-m tall. We recorded measurements from heights of 15.24 cm and 137.16 cm above the nest with the grid at a distance of seven meters away from the nest in each of the four cardinal directions. We quantified nest concealment and vertical structure by taking the percentage of cells per grid (i.e., 200) containing vegetation structure from each location and averaging the four readings in each plot. To measure percent overstory canopy cover, we took digital pictures at 15.24 cm above the ground directly over the nest or plot center depending on plot type. These pictures were then uploaded into the image software program Image J [47] and converted to a binary color format. From this format we calculated canopy cover percentage values by calculating the ratio of black to white pixels within the image.

For overstory basal area measurements, we used a breakpoint DBH (diameter at breast height) of 2.54 cm and defined saplings as trees less than the breakpoint diameter but taller than 1.37 m. Seedlings were defined as those trees less than 1.37 m in height. Within each plot, we identified all trees greater than the breakpoint diameter by species and measured their circumference to calculate the basal area of the plot. For stem density measurements, we counted all sapling stems within the 11.28-m radius plot, and seedlings only within a 3.54-m radius plot [48]. We classified each sapling and seedling as either pine or hardwood.

### Demographic analysis

To calculate summer survival and nest success we used two different analyses. For summer season survival estimates, we used the Kaplan-Meier staggered entry method [49,50] followed by a log rank test to check for differences between 2013 and 2014. This method estimates the probability of an arbitrary animal surviving over specified time intervals (i.e., days) from the beginning of the study [49]. In both years, we extrapolated the rates to reflect a longer, more typical breeding season (i.e., 183-d) [20,51]. The Kaplan-Meier staggered entry method allows for captured bobwhites to be entered over an extended period of time and the data from censored individuals to be used for more accurate estimates. The method required assumptions including random sampling procedures, independent fates, accurate mortality times, homogeneity of survival, attainable consistent locations, and unbiased radio-transmitter effects. Similarly, we used the Mayfield Method [52,53] to calculate estimates of nest success. The Mayfield Method estimates nest success from hatch rates determined from all nests observed, regardless of the amount of time a nest has been incubating at first observation [52]. The Mayfield Method allowed the total number of bird exposure days to be incorporated into final estimates and also for estimates to be generated for nests only partially monitored due to detection in later stages of development. To calculate confidence intervals for the Mayfield estimates we used the procedures outlined in Johnson (1979) [53]. The Mayfield Method followed the assumptions that survival rates were constant over the nesting cycle, all nests visits were recorded, observer effects were inconsequential, successes was measured accurately and every nest exposure day was independent of each other. Though Mayfield estimates are sometimes argued to be biased because of the inability to find bobwhite nests earlier than the incubation period of the cycle; both Mayfield and Kaplan Meier methods are commonly used throughout the quail literature.

In addition to survival analyses, we compared microhabitat characteristics between nest sites and random sites using both descriptive statistics and a principle components analysis (PCA) on 20 variables depicting forest structure. These variables included basal area of overstory trees stratified by pine and hardwood; stem density stratified by pine, hardwood, saplings, and seedlings; percent overstory canopy cover, horizontal cover, vertical structure and percent of ground composition (grass, forbs, woody vegetation, bare ground, and detritus). Aside from the value in comparing means and standard errors, the PCA allowed determination of variables most influential to the variation between nest plots and random plots. Additionally, the PCA biplots provided an illustration of the relationship between plot types and variables. To reduce the number of variables used in the PCA, we created Spearman rank correlation matrices and removed one of each pair(s) of highly correlated variables, keeping the variable with the highest eigenvectors within the first two PC axes. With these results, we created distance biplots to visually compare relationships between variables and among sites.

Lastly, we used telemetry data for each individual with > 24 locations to estimate individual home-range size. For home-range estimates, we used two different techniques: minimum convex polygon (MCP) and two fixed kernel density estimators [31,54]. For kernel estimates, we followed the methods outlined in Janke and Gates (2013) [55] to first compare bandwidth estimators for individual birds in the program Animal Space Use (Version 1.3) [56]; and then used the selected value in the Hawth’s tools extension of ArcGIS (version 9.3, ESRI, Redlands, CA) for the computations. For each individual, the graphical displays in Animal Space Use suggested the least squares cross validation smoothing parameter (LSCV) [57] estimate was the best fit and we therefore used this parameter in Program R. We considered sample size limitations when choosing between the likelihood cross validation (LCV) and LSCV methods for deriving the smoothing parameter [56]. Our data fit the sample size recommendations for number of bird locations used for LSCV ($\bar{x}$ = 77.35 ± 6.07, range = 24–121, n = 20); and locations were adequately dispersed to allow the use of LSCV. For marked individuals that nested during the monitoring period, we used the nest location only once in each of our estimates. Once estimates were calculated, we compared them by sex and year using pair-wise t-tests corrected with Bonferroni adjustments. All estimates of summer demographics (survival and home-range) were based on the seasons 1 April–11 August and 1 April–14 August in 2013 and 2014, respectively. All primary statistical analyses were conducted in the open-source program R (Version 3.0, R Development Core Team 2008, Vienna, Austria).

## Results

### Survival

We captured at total of 66 bobwhites (including recaptures) from 717 funnel traps sites and across 11 mist nets. Based on the number of capture events per trap night, trap success was 0.41% (22 birds / 5165 trap nights) and 2.9% (44 / 1517 trap nights) in 2013 and 2014, respectively (Table 1). All traps were open for an average of 9.3 (range = 0–33) nights. Across both field seasons, we were able to identify six distinct coveys; two in 2013, and four in 2014. Covey size ranged from 6–13 individuals/covey and averaged 9.17 ± 0.95 individuals. Out of nine groups of pen-reared birds released in 2014, one amalgamated with a covey approximately 4-days after release, while another lead us to a wild covey just before mortality occurred also 4-days post-release. Out of the wild birds we were able to detect in 2013, we captured 17 individuals of which only 10 (5 males, 5 females) were fitted with transmitters. In 2014, we captured 21 individuals and radio-marked 19 (7 males, 12 females). Trap predation accounted for the loss of seven individuals across both years while one individual died from trap related injuries. Two of the 17 individuals captured in 2013 were juveniles of unknown sex, and because they weighed < 130g, we did not fit them with a transmitter (Table 1).

**Table 1:** Summary of capture success, survival, and nest success for wild northern bobwhite at Felsenthal National Wildlife Refuge, Arkansas, USA in 2013 and 2014.

	2013	2014
**Captures**		
New	17	21
Recapture	5	23
Total	22	44
**New captures**		
Males fitted with transmitters	5	12
Females fitted with transmitters	5	7
Juveniles	2	0
Trap predation/mortality	5	2
**Right-censored**		
Broken transmitter	2	3
Capture mortality	1	0
Survival past end of study	5	5
**Natural mortality**		
Mammalian predation	1	6
Avian predation	1	2
Snake predation	0	1
Unknown	0	1

Of the ten radio-marked birds in 2013, eight were right censored because of broken collars (n = 2), capture mortality (n = 1); and surviving past the end of the study period (n = 5). In 2014, eight were right censored because of broken collars (n = 3) and surviving past the end of the study period (n = 5). Only one individual was left censored across both years and this occurred in 2014. Mammalian and avian predation accounted for the only two cases of natural mortality in 2013; however in 2014, mammalian predation accounted for 6 out of 10 cases of natural mortality. Avian (n = 2), snake (n = 1), and unknown (n = 1) predation accounted for the other cases (Table 1). In one instance, a radio-marked bird was found dead within a one week period after marking; because it occurred before 1 April 2014, we excluded it from survival estimates.

**Fig 1:**
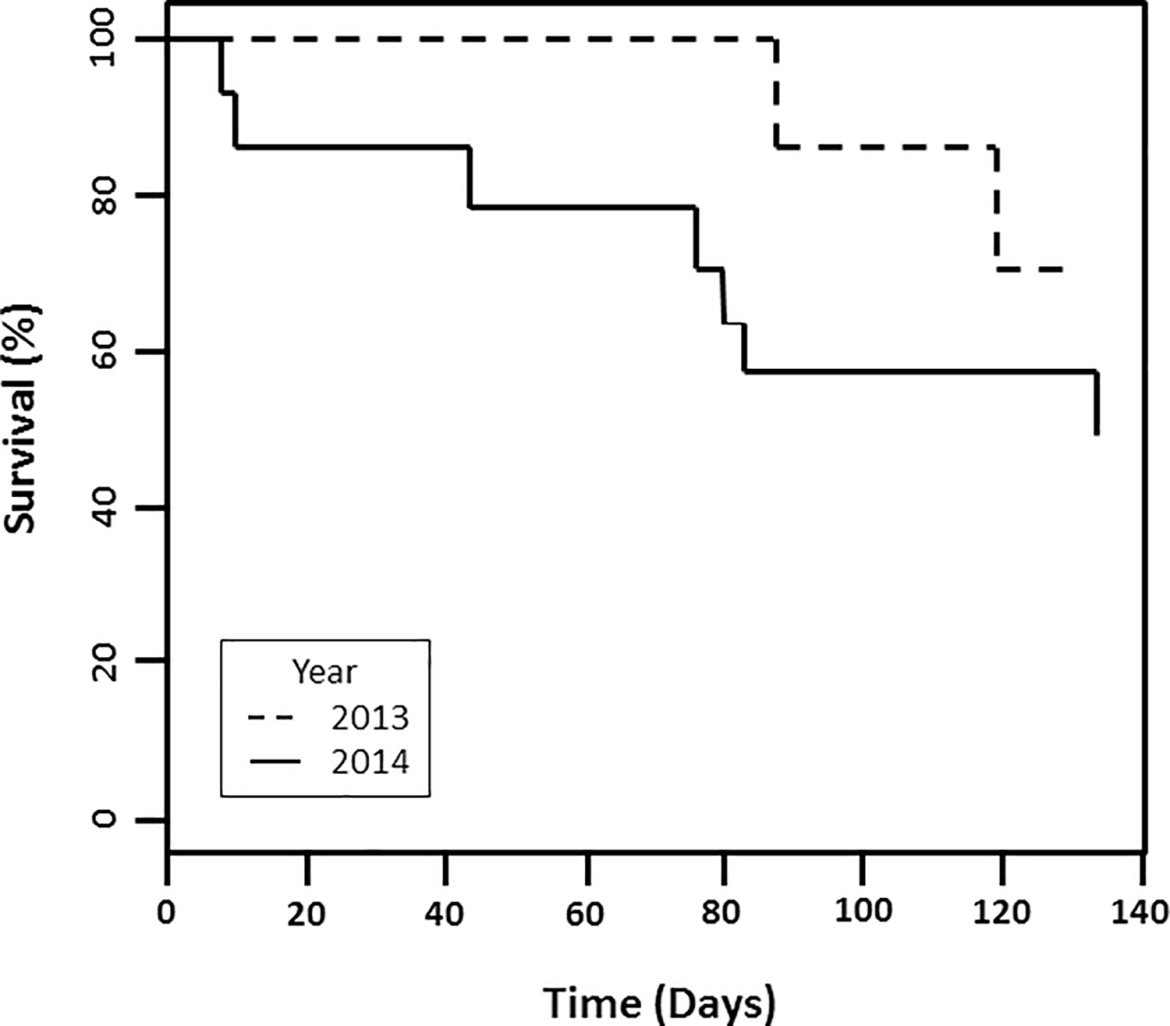
Kaplan-Meier breeding season survival curve for years 2013 and 2014.

Kaplan Meier estimates of summer survival were 0.714 (95% CI = 0.45–1.00) and 0.476 (95% CI = 0.27–0.85) in 2013 and 2014, respectively (Fig 1). Kaplan Meier estimates were based on a 128-day period from 6 April– 11 August in 2013, and a 136-day period from 1 April– 14 August in 2014; and were not different between years (χ_1_^2^ = 1.6, P = 0.21). When we pooled the estimates; 0.502 (95% CI = 0.30–0.83); and extrapolated rates to reflect a 183-d period, the new rates became 0.618, 0.368, and 0.396 for 2013, 2014 and the pooled rate, respectively. While many studies include a 1–2 week acclimation period before including birds in survival estimates, we did not because of the limited field season length and also small sample size [50].

In total, we found ten nests across both years of the study; seven in 2013 and three in 2014. In 2013, two of the five nests we were able to follow the entire incubation period, hatched; whereas in 2014, all three nests hatched. As a result, Mayfield estimates of nest survival were 0.478 (95% CI = 0.201–1) and 1.00 in 2013 and 2014, respectively. Across both years, nests were initiated between 6 May and 26 July and hatched between 3 July and 9 August. In 2013, one nest was found on 6 August and appeared to be in the early stages of incubation; because the field season ended before it hatched we could not document its laying start-date. Across both years, there was only one instance of re-nesting and male-incubation and both occurred in 2013. Mean clutch size for all nests was 14.0 (14.0 ± 0.7, range 9–16, n = 10) eggs and hatchability was 0.86 (51/59 eggs hatched).

Across both years of the study, predation accounted for all failed nests. In 2013 our cameras detected a raccoon destroying one of the nests but failed to document the other two in which we attributed the events to snake predation. Upon visiting these two nests, we found no sign of nest bowl disturbance or missing eggshell fragments. In 2014, the last nest we observed began with a clutch size of 12 eggs, but only ended up hatching one egg upon completion. While monitoring this nest, we observed two events in which 4 and 6 eggs were depredated from the nest within the incubation period. We attributed these events to snakes as well due to the inability of our cameras to detect such instances and also because there was lack of vegetation disturbance at the nest site.

In short, we were only able to completely monitor three broods for brood survival estimates across both years. In 2013, we followed only one brood before the end of the field season and when flushed at 14, 21, 28 and 36 days post hatch, this brood contained only one chick. In 2014, we observed two of the three broods that hatched. Of the older of the two, we failed to observe any chicks on the 14 day flush attempt, but did observe 2 chicks during the 21-d and 28-d flushes. When we attempted to flush the latter brood at 14-d, we observed chicks chirping but could not make a count because of the difficulties in rounding up and flushing the chicks. During the event, the incubating female flushed only a short distance away (approximately 10-m) and proceeded to display distress calls. The field season ended two days after this event and on the last day, the incubating adult’s mortality switch was triggered. We tracked the signal to a mature loblolly pine but could not retrieve the transmitter because it was in the tree’s canopy.

### Habitat

**Fig 2:**
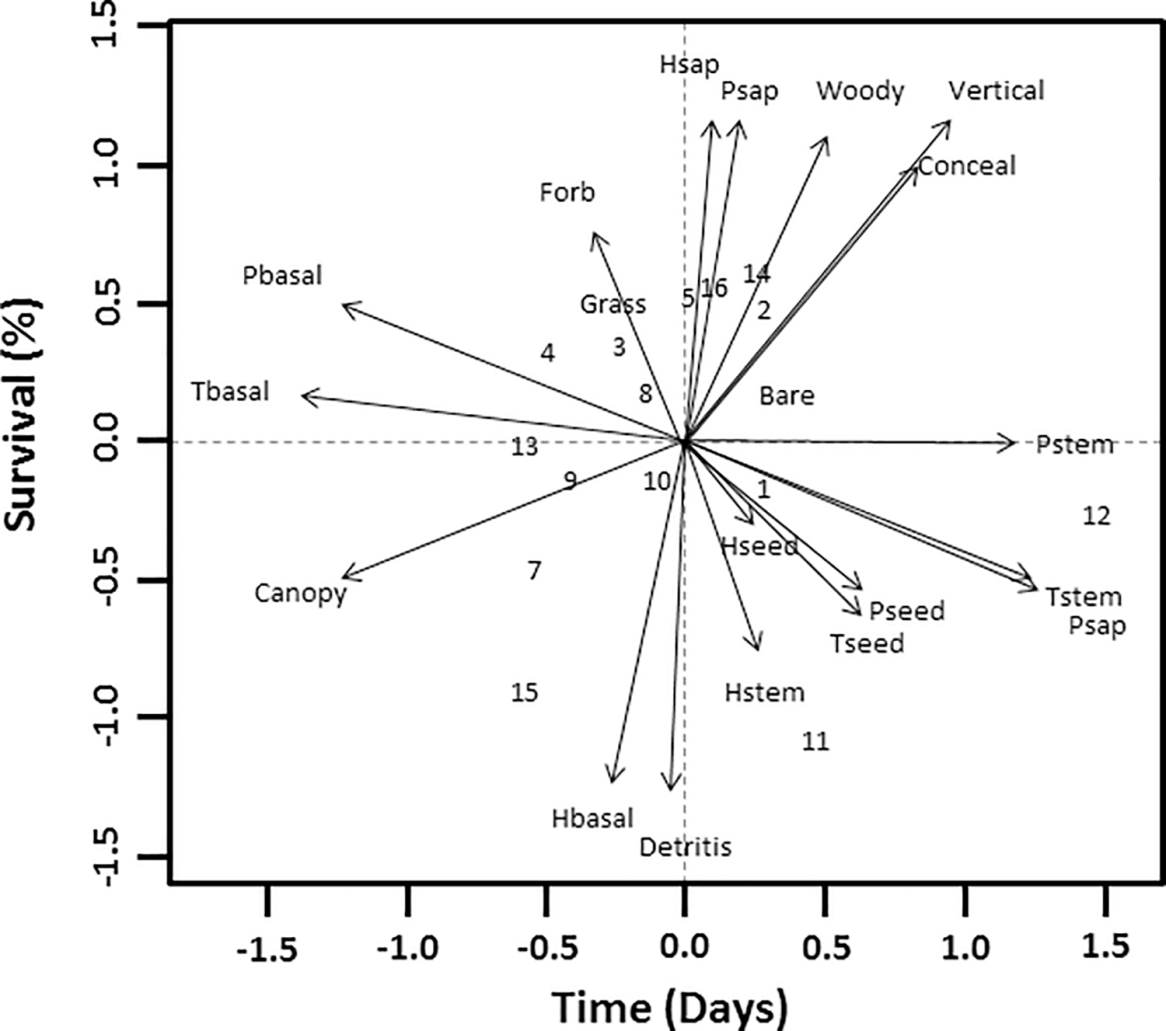
Distance biplot of initial principal components analysis with Scaling 1 for site and species scores. Sites scores are weighted sums of species scores and scaled proportional to eigenvalues. Species are un-scaled with weighted dispersion equal on all dimensions. Variables include Basal Area (Total, Pine, Hardwood), Stem Density (Total, Pine, Hardwood), Sapling Density (Total, Pine, Hardwood), Seedling Density (Total, Pine, Hardwood), Concealment, Vertical Structure, Percent Over-story Canopy Cover, and Ground Cover Composition (Graminoids, Forbs, Woody Plants, Bare Ground, and Detritus). Sites: 1–5 represent successful nests, sites: 6–8 represent failed nests and sites: 9–16 represent random plots.

**Fig 3:**
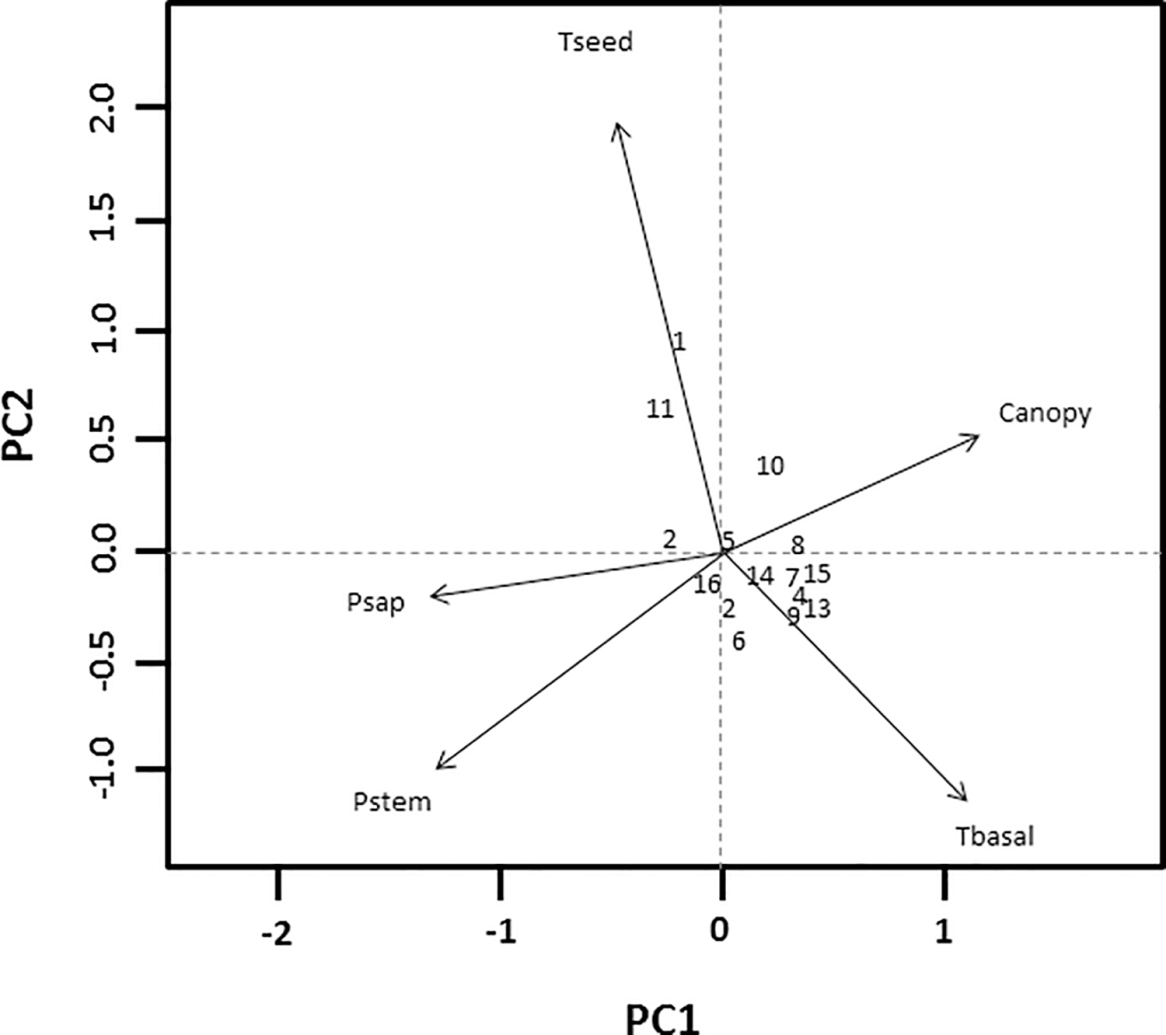
Distance biplot of final principal components analysis with Scaling 1 for site and species scores. Sites scores are weighted sums of species scores and scaled proportional to eigenvalues. Species are un-scaled with weighted dispersion equal on all dimensions. Variables include Total Basal Area, Total Seedling Density, Pine Stem Density, Pine Sapling Density, and Percent Over-story Canopy Cover. Sites: 1–5 represent successful nests, sites: 6–8 represent failed nests and sites: 9–16 represent random plots.

When we initially conducted the PCA with all 20 variables, 44.4% of the variance was explained within the first two principle components and 88.4% within the first six principle components (Table 2). With all 20 variables, broken stick eigenvalues suggested the first 6 axis were the most meaningful. When we reduced the number of variables to five using Spearman correlation matrices, total basal area, pine stem density, pine sapling density, total seedling density and percent overstory canopy cover, explained 81.6% of the variance across the first two principle components (Table 3). Broken stick eigenvalues suggested these two axes were the most meaningful. Out of the remaining five variables, pine sapling density, pine stem density and percent overstory canopy cover fell along the first axis while total seedling density and basal area had the highest eigenvectors along the second axis. Pine stem density along with pine sapling density showed an inverse relationship with percent canopy coverage as did total seedling density and total basal area. With the exception of three random plots and one successful nest plot, most plots (both random and nest) appeared relatively clumped along the central vertex (Fig 2) and the eigenvector representing total basal area (Fig 3).

**Table 2:** Importance of components of initial principal component analysis and species scores.

	Principle Components	
	1	2
**Eigenvalue**	4.69	4.18
Standard Deviation	2.17	2.05
Proportion Explained	0.23	0.21
Cumulative Proportion	0.23	0.44
**Eigenvectors**		
Total Basal Area	-1.63	-0.20
Pine Basal Area	-1.44	-0.61
Hardwood Basal Area	-0.31	1.43
Total Stem Density	1.47	0.59
Pine Stem Density	1.36	0.01
Hardwood Stem Density	0.32	0.93
Total Sapling Density	0.21	-1.33
Pine Sapling Density	1.51	0.66
Hardwood Sapling Density	0.06	-1.36
Total Seedling Density	0.81	0.75
Pine Seedling Density	0.75	0.65
Hardwood Seedling Density	0.27	0.34
Canopy	-1.39	0.54
Concealment	0.93	-1.14
Vertical Structure	1.14	-1.39
Grass	-0.28	-0.53
Forb	-0.41	-0.91
Woody	0.60	-1.28
Bare	0.27	-0.16
Detritus	-0.06	1.48

**Table 3:** Importance of components of final principal component analysis and species scores.

	Principle Components	
	1	2
**Eigenvalue**	2.85	1.23
Standard Deviation	1.69	1.11
Proportion Explained	0.57	0.25
Cumulative Proportion	0.57	0.82
**Eigenvectors**		
Total Basal Area	1.26	-1.28
Pine Stem Density	-1.48	-1.12
Pine Sapling Density	-1.57	-0.23
Total Seedling Density	-0.57	2.30
Overstory Canopy Cover	1.45	0.63

When we compared the microhabitat variables by their means and standard errors, only the percent ground cover of forbs and detritus between successful nests and failed nests differed (Table 4). The percentage of forb cover was nearly six times higher while the percentage of detritus was almost twice as low at successful nests compared to failed nests. In general, nest concealment and percent grass, forb and woody cover were all higher at nests and successful nests compared to random plots and failed nests, respectively. Successful nests had lower overstory tree stem density (i.e. total stem density) than failed nests, but higher sapling density (successful $\bar{x}$ = 93.6 ± 17.3, successful; $\bar{x}$ = 86.7 ± 27.1, failed). Successful nests had lower basal area on average than failed nests (Fig 3; Table 4).

**Table 4:** Means and standard errors of vegetation measurements at 0.04 ha circular plots at the nest location (n = 8), random points (n = 8), successful nests (n = 5), and failed nests (n = 3).

	Nest		Random		Successful		Failed	
Variable(s)	Mean	SE	Mean	SE	Mean	SE	Mean	SE
Pine Basal Area (m^2^/plot)	0.7	0.1	0.6	0.1	0.6	0.2	0.8	0.1
Hardwood Basal Area (m^2^/plot)	0.0	0.0	0.1	0.0	0.0	0.0	0.0	0.0
Total Basal Area (m^2^/plot)	0.7	0.1	0.7	0.1	0.6	0.2	0.8	0.1
Pine Stem Density (#/plot)	5.4	1.3	10.3	5.4	4.4	1.0	7.0	3.2
Hardwood Stem Density (#/plot)	4.3	1.6	7.8	3.4	3.2	1.2	6.0	4.0
Total Stem Density (#/plot)	9.6	2.7	18.0	5.1	7.6	1.3	13.0	7.2
Pine Saplings (#/plot)	0.8	0.6	3.9	2.8	1.0	1.0	0.3	0.3
Hardwood Saplings (#/plot)	90.3	13.5	69.4	29.2	92.6	16.8	86.3	27.4
Total Saplings (#/plot)	91.0	13.7	73.3	28.5	93.6	17.3	86.7	27.1
Pine Seedlings (#/plot)	48.3	39.3	57.9	29.4	68.4	63.3	14.7	12.7
Hardwood Seedlings (#/plot)	38.9	11.7	40.3	13.9	51.8	16.3	17.3	5.2
Total Seedlings (#/plot)	87.1	39.0	98.1	33.4	120.2	58.8	32.0	16.3
Graminoid (%)	13.7	1.6	12.6	2.0	14.9	1.9	11.5	2.9
Forb (%)	9.7	2.6	4.5	1.2	14.0	2.6	2.4	0.1
Woody (%)	31.9	4.7	26.8	3.3	36.3	4.7	24.5	9.2
Bare (%)	3.1	1.2	4.8	1.2	3.2	2.0	3.0	0.3
Detritus (%)	49.9	7.2	55.8	6.1	38.7	7.4	68.6	4.9
Vertical Structure (%)	0.7	0.0	0.7	0.1	0.7	0.1	0.7	0.1
Nest Concealment (%)	1.0	0.0	1.0	0.0	1.0	0.0	1.0	0.0
Tree Canopy Cover (%)	0.5	0.0	0.5	0.1	0.4	0.0	0.6	0.1
Tallest Vegetation Height (m)	1.0	0.2	0.8	0.1	0.7	0.2	1.4	0.5

### Home range

For each of the home-range estimates, there was an average of 77.4 (range = 24–121) locations per individual. All but one individual had > thirty locations; therefore sample size did not influence either 95% kernel estimates (P = 0.239, r^2^ = 0.0248), 50% kernel estimates (P = 0.167, r^2^ = 0.536) or MCP estimates (P = 0.596, r^2^ = -0.0387). Of the three estimates, only the MCP estimates across years differed (Table 5). MCP estimates in 2013 ($\bar{x}$ = 113.8 ± 20.1) were lower than those in 2014 ($\bar{x}$ = 393.1 ± 49.0; P < 0.001). Mean 95% kernel home-range size was 63.9 ha (95% CI = 48.7–79.2) and mean 50% kernel home-range size was 14.9 ha (95% CI = 11.28–18.6) pooled across years, respectively.

**Table 5:** Mean home-range estimates and standard errors (SE) of male and female radio-marked northern bobwhite and radio-marked northern bobwhite in years 2013 and 2014.

	Male		Female		2013		2014	
Home-range type	Mean	SE	Mean	SE	Mean	SE	Mean	SE
95% Kernel (ha)	68.0	12.6	59.8	7.8	64.4	16.1	63.6	6.5
50% Kernel (ha)	15.8	3.0	14.1	1.9	15.9	3.9	14.3	1.5
MCP (ha)	220.2	42.2	342.6	73.0	113.8	20.1	393.1	49.0

Only six distinct coveys were identified across both field seasons despite extensive searching, calling, trapping, and the release of pen-reared bobwhites. Within our study area, distance between coveys ranged from 1.42− 4.29 km and their size and movements indicated they were likely the only coveys in the area (J. Doggett, personal observation). Past research has illustrated that distance between coveys increases as density decreases [58]; thus, the large distances in our study suggested the number of individuals on the refuge was indeed small and reflective of a low-density population [59].

In a study area of approximately 3,000-ha in size, six coveys equates to 0.0020 birds/ha; which is considerably low compared to very low densities reported in Ohio (0.0025−0.0163 birds/ha) [59]. Other research reported densities [60–63] ranging from 0.012−0.58 birds/ha and reported an average distance of 0.96 km between radio-marked coveys in highly fragmented habitat.

## Discussion

The apparent overlap in management practices between RCWs and bobwhite [12,13,64] suggest that intensive RCW management at Felsenthal NWR should create favorable conditions for bobwhite; however, the number of birds observed in our study despite significant trapping effort suggested the population on Felsenthal NWR is very low. Factors affecting population growth may include nest success (i.e. the production of fledging offspring), low brood survival and winter survival, poor habitat conditions, and population isolation. Several of these factors are mechanisms of recruitment.

Estimates of nest success were comparable (in 2013) or higher (in 2014) to those reported in Texas (49%) [65], New Jersey (45.4%) [66]; Florida (41%) [67], and Kentucky (31.7%) [68]. Average clutch size on Felsenthal NWR was higher than the assumed range-wide average of 12 eggs [1]; and, with the exception of the hatchability rate in 2013; (77%), the pooled hatchability rate (86%) was in range compared to rates reported in the literature (80% - 96%) [20].

Nest site characteristics were different and typically less variable than random sites (Table 4). Bobwhites appeared to select open areas in the forest with > 30% woody understory vegetation and a predominance of woody, forb, and grassy ground cover (Table 4). Compared to descriptions of nesting habitat in the literature, the characteristics of nest sites within our study area seemed in line with what others have reported. In Oklahoma, there was greater woody cover at nests (20–30%) compared to random sites (10–15%) [69] while in Texas, nest sites and successful nests had greater percentages of shrub and bare ground exposure and also taller vegetation height over nests in order to provide concealment [35]. Considering the high rates of nest success, nesting habitat likely is not limiting for bobwhite at Felsenthal NWR.

Estimates of summer survival were within an acceptable range of a growing population and were comparable to summer survival estimates in studies of larger populations. With the exception of the 2013 estimate, our estimates were about average compared to others in the literature; 25.3% and 27.9% in Kentucky [70], 33.2% in Missouri [22], 33% in North Carolina [23], and 34.3% in New Jersey [71]. The adjusted pooled rate of 39.6% was considerably lower than an estimate by Sandercock et al. (2008), who showed using life-stage simulation analysis, a summer rate ≥ 79% would be required to support a growing population [20]; however, the estimate was relatively good compared to Sisson et al. (2009) who showed using long-term data; even a summer rate of 35% could support a growing population [72]. Both studies complimented their estimates with winter survival rates of ≥ 50%, which is recommended for accurate population growth rates [20,73]. Considering the small sample size, summer survival alone appeared sustainable on Felsenthal NWR.

Because our sample size was small and small samples can bias survival estimates [50], we analyzed our estimates of summer survival using another set of criteria [51]. Within the bobwhite literature, some researchers argue telemetry based survival estimates are biased low do to the potentially negative effects of radio-transmitters [51]. In particular, the authors suggested that for telemetry-based survival estimates to be realistic, they should represent a juvenile: adult age ratio less than 7:1 [51]. A 7:1 age ratio has been used to represent the maximum reproductive potential theoretically possible for northern bobwhite such that any ratios higher then 7:1 exceeds the limits of bobwhite reproduction [51]; but also, age ratios < 4 are typically considered low and inadequate for population growth [63]. We assessed our rates based on telemetry assumptions and determined theoretical age ratios they would consider reasonable: 1.61:1 in 2013; 6.33:1 in 2014 and 5.36:1 for the pooled rate. Our calculated ratios were below 7:1 which was good because they suggested summer survival on Felsenthal NWR was acceptable for a sustainable population, and thus, likely not directly causing the population decline.

Our data suggested that the low numbers of bobwhite observed may be due to a problem with brood survival (i.e., recruitment). Compared to brood survival estimates theoretically required to sustain a population [51,63], it was apparent that brood survival may be too low on Felsenthal NWR. Across the literature, brood survival ranges from 0.14–0.72 [20] and is typically regarded as the least understood aspect of bobwhite ecology; however, low brood survival directly translates into low fall recruitment and low recruitment can significantly impact a bobwhite population [19,20]. A lack of brood production on the study area could be impacting fall population size and consequently reducing population growth rates.

Other factors could also be contributing to low recruitment on Felsenthal NWR. For bobwhites, the ability to have multiple broods throughout the breeding season and the propensity to re-nest after failed attempts is thought to be a mechanism of recovery after years of low annual survival [22,74]. Theoretically, if bobwhites nested later in summer such instances could reduce recovery potential via a shortage of nests and surviving chicks [75,76] because the total number of nests built in a breeding season was a good predictor of fall density [24]. On Felsenthal NWR, nest initiation though more typical in 2013, was relatively later than reports of first nest initiation in the literature, especially in 2014. Clutch initiation reportedly occurred as early as 16 April in southern Illinois [21]; and even earlier in Georgia and Texas [77,78]. Peak nest initiation was typically associated with the end of May and first two weeks in June [21,77,78], but at times may occur at the end of April [79]. Based on the nests we observed during the assessment, first nest initiations ranged between 6 May and 27 June in 2013; but between 3 June and 4 July in 2014. Though it is possible the weather may have impacted nesting in both years, we did not observe or find in the record any extreme climatic patterns that would appear to have been influential [80]. In fact, average monthly temperatures from between April and September in southern Arkansas did not differ by more than 1° C in all months except June, which differed by 2.8° C [80]. Precipitation was also similar in both years. Nest predation is by far the most common cause of nest failure [40,81], and prior to incubation usually goes unnoticed in telemetry studies; however, if nesting was indeed delayed it may be responsible for the lack of broods we observed before the end of each field season.

In addition to late nest initiation, covey break up on Felsenthal NWR seemed unusually late especially in 2014. Though rarely discussed in the literature, late covey break up is intrinsically linked to nest initiation dates. On Felsenthal NWR, covey break up ranged from 15 April−15 May and was considerably later in 2014 than in 2013. For bobwhites, spring pair-bonding is facilitated when suitable mates are within the covey prior to break up, and supplementary covey mixing during winter could facilitate earlier nest initiation [78]. A limited number of breeding pairs on Felsenthal coupled with low annual recruitment could theoretically result in the need to disperse longer distances to find suitable mates [82]. Distances between coveys in our study area were larger than other estimates within small populations [63,83,84], suggesting that bobwhites needed to travel further than average to find suitable mates, which created several problems for population growth.

Some researchers postulated that the negative impacts of large distances between coveys could decrease survival during the non-breeding season and impact population growth rates [59]. Other reports suggest that as distance increases between coveys in low density populations [58], so does the infrequence of individual transfers between coveys [56,85]. Even further, [86] reported that individual survival tends to decrease as covey size fluctuates above or below an optimal size of 11 individuals. On Felsenthal NWR, mean covey size during March and April was only 9.2 individuals and lower than reported averages [1]. Thus, large distances could have inhibited the transfer of individuals among coveys on Felsenthal NWR and resulted in covey sizes below the optimal level. If optimal covey size was higher than 9.2 individuals on Felsenthal NWR, bobwhites were likely experiencing reduced winter survival [86]. Reduced winter survival translates into a smaller breeding population and reduced recruitment potential.

Large distances between coveys likely affected breeding season home range size. Breeding season home range size (63.9 ha) exceeded many estimates for 95% kernel distributions; 21-ha in Georgia [87]; 38-ha in New Jersey [71]; 54-ha in Florida [88]; and 74-ha in Kansas [46]. Home ranges in areas closer and more similar to Felsenthal NWR had a 95% kernel distribution estimate of 61.9-ha in the pine forests of East Texas [14] and 58.4-ha in Louisiana [89]. In both of those studies, authors suggested their home-range estimates were large mostly because of the poor quality of pine-forest ecosystems in general. Thus, estimates from observations on Felsenthal NWR were relatively high considering the presence of quality habitat due to conditions created from RCW management [12,13,26,64].

Our observations suggest that the isolated characteristics of the refuge in general, better explain the movement patterns of radio-marked bobwhites than poor habitat quality. Many researchers would agree that bobwhite movements are dictated by habitat quality [39,90] such that limited resources increase home-range size [91], however; the degree to which the site is fragmented from other suitable sites may also decrease home-range size and prevent dispersal [56,87]. Researchers documented decreased covey movements in fragmented habitat and suggested the availability of suitable habitat may have restricted movements [56]. Since none of the radio-marked birds in our study were lost due to excessive movements off the refuge, it might be naïve to conclude that habitat quality in the study site was indeed poor [92], but rather, dispersal off the refuge appeared to be restricted. The possibility of restricted movement off of the refuge strengthens the possibility that the population may be isolated and those dynamics could explain the low density characteristics we observed [56]. If the population was isolated or immigration to the refuge was minimal, the population could be experiencing problems associated with reduced gene flow [93]. During the study, we failed to observe bobwhites dispersing large distances off of the refuge. Several of the 2014 radio-marked individuals did disperse to adjacent non-federally owned properties which were also managed for RCW’s, and stayed there for the duration of the summer. However, these properties were juxtaposed uniquely along the refuge boundary and surrounded by other property that was intensively managed for timber (J. Doggett, personal observation). Except for short periods of time, radio-marked birds did not disperse beyond the boundaries of the RCW managed areas such that the limits of the property represented the furthest distances away from the refuge radio-marked birds traversed.

In addition to low juvenile recruitment and population isolation, winter survival could explain the population dynamics of bobwhite on Felsenthal NWR. We did not quantify winter survival, but future research should record information on winter survival to understand a clear picture of annual population dynamics. For example, compared to summer survival and brood survival, winter survival has been shown to contribute considerably to variation in rates of population change [19,20,86]. If nesting and brood survival increased significantly the last month of the breeding season and high winter mortality significantly reduced the number of individuals entering the breeding season, low winter survival could be a reasonable explanation for the low-density population on Felsenthal NWR. Low winter survival is typically associated with severe weather, a decline in habitat availability, food shortages, or increased predation rates.

Future research should also strategically evaluate specific habitat conditions for bobwhite on the refuge to identify areas that may need improvement to ensure connectivity between patches of suitable habitat. Coordination of conservation efforts with adjacent landowners is essential to maintain habitat conditions facilitating breeding and survival [94]. This area of the West Gulf Coastal Plain is ranked as having medium potential for bobwhite restoration and conservation [95]. Understanding the underlying reasons for population declines in an area will help conservation agencies and organizations target specific habitat features affecting survival. This understanding is critical for promoting establishment and enhancement of contiguous bobwhite habitat in the West Gulf Coastal Plain and throughout the bobwhite’s range.

## Conclusions

Researchers estimate that approximately 11% of the land area in the WGCP contained habitat suitable enough to support densities of at least 0.14 birds/ha [2], which is the recommended restoration goal of the National Bobwhite Conservation Initiative [96]. Even further, they also showed only 8% of the land cover within the WGCP supported sustainable populations of 400–700 individuals [2]. Given that their model was based on land cover data from the early-to-mid 1990’s, trends in the WGCP continue to decline and bobwhite populations in Arkansas likely face similar constraints today [1]. Our data suggest the bobwhite population at Felsenthal NWR is declining due to low recruitment from brood survival and a high degree of isolation between other local bobwhite populations.

Resources for restoration efforts should not be allocated in areas where populations are below sustainable levels while others [97], recommend restoring areas near already suitable habitat to increase local abundance [93]. Since management for RCW already occurs on Felsenthal NWR, we suggest developing management plans to increase connectivity between areas managed for RCW and those in surrounding landscape that have potential to be good bobwhite habitat. As bobwhite numbers continue to decline across the WGCP, concerns of population extirpation will likely become more prevalent and management actions to increase dispersal among isolated populations will be needed. Working with private landowners to increase habitat quality on land adjacent to Felsenthal NWR could increase population growth in the landscape and alleviate concerns of isolation between populations of bobwhites, especially areas already in close proximity [93,96].

Finally, we recommend initiating research that directly addresses the management discrepancies between RCW and bobwhite, perhaps more specifically, research that investigates the dynamics within a loblolly pine-dominated landscape. Goals for managing RCW in different forest types are often site-specific and understanding how those different management practices affect bobwhites would undoubtedly answer detailed questions about habitat quality for both species. Since bobwhites are declining range wide and among different habitats, we suggest areas like Felsenthal NWR will become increasingly more important to restoring bobwhite populations across their range, in particularly the WGCP where declines are severe and regional conservation goals are unique.

## Supporting information

S1 File Telemetry data for northern bobwhite quail in Felsenthal National Wildlife Refuge, Arkansas, USA during 2013–2014. (ZIP)
